# Relationship between Emotional Intelligence and Violence Exerted, Received, and Perceived in Teen Dating Relationships

**DOI:** 10.3390/ijerph18052284

**Published:** 2021-02-25

**Authors:** Cordelia Estevez-Casellas, Mª Dolores Gómez-Medina, Esther Sitges

**Affiliations:** CRÍMINA Center for the Study and Prevention of Delinquency, University Miguel Hernández, 03202 Elche, Spain; c.estevez@umh.es (C.E.-C.); mdgomez@crimina.es (M.D.G.-M.)

**Keywords:** dating relationships, violence, emotional intelligence, adolescence

## Abstract

Emotional intelligence plays a critical role in adolescence since it involves a change towards psychological, social, and sexual maturity; a stage in which the foundations of intimate social relationships are established. Emotional competences regulate the quality of these relationships in adolescence and can provide protection against or facilitate the use of violence within them. Based on the above, this study aims to analyze the relationship between emotional intelligence and violence exercised, received, and perceived by adolescents in dating relationships. A sample of 254 subjects (43.1% men and 56.9% women) between 12 and 18 years old was analyzed through the Emotional Intelligence Questionnaires of BarOn ICE:NA and Violence Exercised Perceived and Received by Adolescents VERA. The results of the research have shown that there is a significant and inverse relation between the dimensions of emotional intelligence and the violence exercised by adolescents in their dating relationships, and a positive and significant relation between emotional intelligence and the perception of violent behavior. For this reason, the importance of educating people about emotional intelligence from childhood within both the academic and family sphere is highlighted. This is fundamental to preventing the appearance of such violent behaviors and promoting an adequate adaptation to the environment.

## 1. Introduction

Adolescence is a stage when important physical, psychological and cognitive changes take place. The high emotional intensity and social significance of these changes provides a context where friendships with peers play a significant role in the development of emotional balance. During this stage, adolescents build up long-lasting and affectionate relationships of awareness, loyalty, sincerity, intimate communication, and prosocial behaviors. Through these relationships with peers, young people seek to build their identity: a sense of belonging as well as sharing lifestyles and emotional empathy [1,2,3,4,5,6]. 

The first dating relationships and sexual experiences typically start during this stage of life. In this respect, different authors have shown that there is a link between patterns of violent behaviors in the dating relationships adolescents establish and their inexperience in partner relationships, their difficulty in perceiving certain violent behaviors as aggressions, and/or their social learning about maladaptive behaviors in adults [7,8,9].

In fact, in developing countries, 1 in every 4 women and 1 in every 5 men say that they have suffered and used violence and physical abuse in an intimate relationship [10,11]. Similarly, the World Health Organization Report (WHO) confirms that victims of partner violence are more prevalent in adolescents aged between 15 and 19 and young adults aged between 20 and 24 (29.4% and 31.6%, respectively) than in women aged between 55 and 59 [7,12].

This problem of violence in teen dating is considered an issue of great interest in scientific research. In some studies, it has been observed to be bidirectional but not necessarily symmetrical; that is to say, there are women who assault their male partners, violence between same sex couples, and a prevalence in violence by men towards their female partners [13,14,15,16,17,18,19].

Although the conclusion reached in different studies is that both men and women display all these behaviors, it is also noted that there are differences between how they are manifested in each sex. For example, it has been found that women use psychological violence to a greater extent, while physical and sexual violence is used more frequently by men, and in this case, it is women who are the victims of these aggressive acts [20,21,22,23,24,25,26,27,28,29].

As far as cyber violence is concerned, all studies agree that over the last few decades this type of aggression has increased at the same time as advances in technology and the internet have been made. Studies on adolescents reflect that 27.2% of participants had used impersonation to control their partners and 59% had tried to control interaction through their partners’ social networks [20].

All these acts of violence lead to immediate consequences for victims, especially adolescent partners, who are mainly affected on an emotional level. Some of the effects include anxiety, stress, low satisfaction with life, low academic performance, depressive symptomatology, toxic substance abuse (tobacco, alcohol, marijuana, and other drugs), low self-esteem, anti-social and risky sexual behaviors, eating disorders, or suicidal ideation. [7,13,30,31].

Furthermore, a large amount of scientific literature agrees that there are risk factors that increase the likelihood of these violent behaviors appearing in adolescent couples. These include toxic substance consumption, having been a victim of or having witnessed violence at home, consumption of alcohol and drugs, difficulty in regulating emotions, low self-esteem, positive attitudes towards the use of violence, and sexist beliefs [7,13].

For this reason, the emotional aspects of this stage of the life cycle are considered to be determining factors for individuals’ adaptation to their environment. In this sense, greater importance is being given to research on the influence of Emotional Intelligence in different areas such as violence, emotional adjustment, sexual relations, attitudes, motivation, etc.

In fact, different studies show that there is an inverse relation between different components of Emotional Intelligence and the violence exercised by adolescents and the quality of their affective relationships. Those who show higher levels of behavioral problems and anti-social behaviors (aggressiveness, negative thoughts about others and about life, threats, insults, delinquency, etc.) are less able to self-manage their emotions or have inadequate emotional management, and have difficulties in self-control and empathy; that is to say, they generally show low scores in Emotional Intelligence [32,33].

For this reason, the main aim of this research is to analyze the relation between Emotional Intelligence in adolescents and the violence they exert, receive and perceive in their dating relationships. The specific objectives are outlined below:

1. To analyze whether there are significant differences in Emotional Intelligence between boys and girls.

2. To analyze whether there are significant differences in the violence that they use according to gender.

3. To analyze whether significant differences can be found in the violence adolescents receive in their dating relationships according to gender.

4. To analyze whether there are significant differences between boys and girls when identifying violent behaviors in dating relationships.

5. To analyze whether there are significant differences in the dimensions of violence perceived between those who have had a dating relationship and those who have not.

Based on the information found in the bibliography and for all these objectives, we believe that Emotional Intelligence will be a protective factor against exercising or receiving violence in adolescent dating relationships, as well as helping adolescents to be more skilled at identifying violent behaviors used by their partners.

## 2. Materials and Methods

The sample comprised of 254 adolescents from La Torreta Highschool (Elda) who are currently in the 1st, 2nd, 3rd, and 4th year of Secondary Education; 43% were boys and 57% were girls aged between 12, and 17.67% of this sample indicated that they had had a dating relationship that lasted exactly one month or more, while 33% reported that they had had no relationships.

In order to evaluate violence exercised or received by adolescents, the inclusion criteria were participants whose relationship/s had lasted a minimum of one month. To evaluate perceived violence, the whole sample was used regardless of their characteristics or situation.

Before carrying out the study, first an appointment was made to visit the school and explain what the study consisted of and ask for their collaboration and the necessary permission. Next, after receiving confirmation of participation, the school guidance counselors were contacted to organize timetables for the questionnaires to be administered to the minors, provide all the documents, and explain how each questionnaire should be completed (informed consent, the V.E.R.A questionnaire [34], and the Emotional Intelligence Inventory Abbreviated BarOn ICE: NA [35]).

Before completing the questionnaires, the informed consent form was given out to students, which explained what the research was about and who was responsible for it. In order to participate in the research, the minors had to be interested in participating, had to hand in the informed consent signed by their father/mother or guardian, and the minors themselves had to approve the conditions of the study.

The questionnaires were administered in groups in school classrooms during tutorial periods to avoid disrupting timetables for main subjects. They were answered individually by the students who had previously handed in the informed consent signed by both parents/legal guardians and themselves, indicating that they agreed to participate in the study. The time required for the explanation and completion of the questionnaires was 50 min, although this time varied for different school years; students from the third and fourth year required less time.

Once the evaluation was done, all the data collected was entered into the statistical software SPSS 22.0 (Armonk, NY, USA), which was used to obtain all the analyses and results for the research through correlation analysis and comparison of means for independent samples.

The socio-demographic variables used in this study were: age, school year, sex, number of partners they have had, and duration of the longest relationship. All data for these variables were collected through open questions, which participants had to answer fully.

The dependent study variable was violence exerted, received, and perceived by adolescents in dating relationships. To gather this data, we used the Questionnaire of Violence Exerted, Perceived and Received by Adolescents VERA [34]. This is a self-report instrument, where minors respond to questions about: the violence they have used/use against their partner/s, they have received/receive in their dating relationships, and how violent they perceive the behaviors presented in the questionnaire to be. These behaviors are presented through 28 items that encompass information about five types of violence.

*Physical Violence* (items 1, 6 14, 17 and 20), understood to be any non-accidental act that causes or can cause physical harm, injuries, or even the death of a person subjected to this violence (hitting, pushing, throwing objects, biting, strangling, beating, scratching, spitting, burning, using objects or part of the body to threaten and overpower a person, etc.).

*Sexual Violence* (items 7, 8, 9, 12, 21 and 24) is defined by the WHO [36] as behaviors that force a person to maintain sexual relations against their will or sexual relations that can be humiliating or degrading that are maintained because of fear of what their partner might do (sexual denigration, treating the victim as an object, fondling, abuse, assaults, being coerced into prostitution etc.).

*Social Psychological Violence* (items 3, 5, 13, 16 and 25): This refers to those behaviors that directly attack and harm the victim’s social and family relationships (isolation, phone-call restriction, etc.) and aim to prevent the victim from being able to have other points of view, or from asking for or getting help.

*Psychological Violence of Control* (items 2, 10, 15, 19, 23 and 27): This includes any behavior and demands that aim to control an individual’s behaviors and relationships and prevent the victim from being able to do other activities.

*Psychological Violence of humiliation* (items 4, 11, 18, 22, 26 and 28) defined as acts and behaviors that aim to unfairly debase and ridicule a person or group of people through insults, threats, etc., causing a person to suffer negative feelings (embarrassment, agitation, sadness etc.).

This questionnaire has a Likert scale with six response options for the items that measure violence exerted and received (0 = never, 1 = once, 2 = from 2 to 5 times, 3 = from 6 to 10 times, 4 = from 11 to 15 times, and 5 = more than 15 times); and a 5-option response scale to measure perceived violence (0 = not violent, 1 = only a little violent, 2 = quite violent, 3 = considerably violent and 4 = very violent).

Its psychometric properties show adequate values of confidence in the five dimensions with a Cronbach’s alpha of 0.99 and a correct construct validity.

The independent variable Emotional Intelligence is measured using the abbreviated version of the Inventory for Emotional Intelligence BarOn Ice, which is aimed at a population aged between 7 and 18 [37]. This scale comprises of 30 items that are answered on a 4-point Likert scale from 1 (very rarely) to 5 (very often). These items encompass information from the 6 scales of Emotional Intelligence based on the Bar-On theory [38]: *Interpersonal Intelligence* (to evaluate empathy skills, interpersonal relations, and social responsibility); *Intrapersonal Intelligence* (to measure aspects like emotional self-awareness, assertiveness, self-concept, self-fulfillment and a positive vision of oneself); *Adaptability* (problem solving skills); *Stress Management* (to evaluate an individual’s stress tolerance and impulsivity); *Positive Impression* (a control variable that measures a highly favorable perception of oneself; that is to say, the social desirability an individual displays in the questionnaire); and *Total Coefficient of Emotional Intelligence* based on all the above-mentioned dimensions.

In this case, it is highly important to consider the score obtained in the control dimension of positive impression, since high scores would invalidate the test as it would show a high social desirability in the subjects, so the data obtained would not be representative of the sample. Therefore, an adequate score in this dimension, together with adequate psychometric properties, where there is a high Cronbach’s alpha of 0.81, would show that it is an adequate instrument for measuring emotional intelligence in minors.

This is a cross-sectional study, which follows a non-experimental or descriptive quantitative method, where questionnaires are used to evaluate the characteristics and variables of interest.

In this research, the qualitative variables observed are sex and whether a dating relationship has been maintained. The quantitative variables measured are: age, the emotional intelligence values obtained (independent variables), and the extent of the violence received, exercised, and perceived in dating relationships (dependent variables).

The statistical analysis carried out in this research is descriptive so as to obtain a profile of the subjects in the sample (sex, age, dating relationships, the amount of violence exerted, received and perceived, emotional intelligence values etc.).

In addition, a Pearson correlation analysis was performed to analyze whether there is a relation between the dimensions of emotional intelligence and violence exerted, received, and perceived in adolescents. Finally, three student’s T-tests were used to make comparisons between the two independent samples. The first one is to compare whether there are differences between men and women in the violence they exercise, receive, and perceive. The second one is to verify if differences can be found in the values obtained in the different dimensions of emotional intelligence of men and women. The last one is to observe whether there are significant differences in the perception of the different behaviors according to whether a dating relationship has been/is maintained or not.

It is important to take into account that the analysis of the results will be carried out based on an α of 0.05, and all data below this coefficient will be considered significant.

## 3. Results

**Figure 1:**
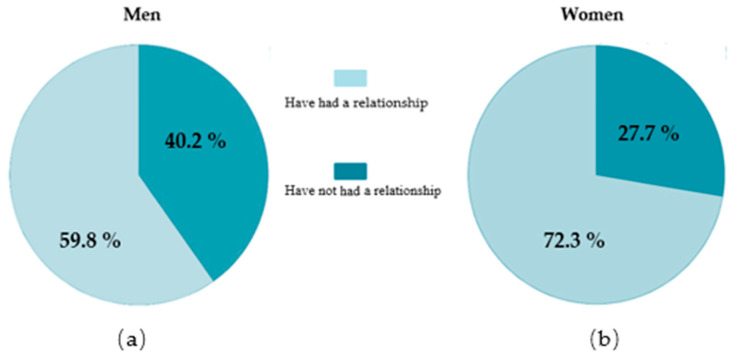
Percentage of adolescents who have had/have a dating relationship according to sex. (**a**) Percentage of male adolescents who have had/have a dating relationship; (**b**) Percentage of female adolescents who have had/have a dating relationship.

The graphs in Figure 1 illustrate the sample used for this research. 67.3% of the participants indicated that they have had at least one dating relationship, with a higher percentage of girls having had more than one relationship in contrast to boys.

In relation to the descriptive analyses, in the Table 1, of the emotional intelligence variable, we found the sample obtained mean scores below the mean score of the abbreviated version of the Bar-On Ice: NA; therefore, all scores in all the dimensions of Emotional Intelligence are low. One point of interest in these results is the value obtained in the control variable of positive impression. As it is low, it shows that the desirability reflected by subjects in the questionnaire is low, and consequently the scores in the Emotional Intelligence dimensions fit the participants’ profile.

On the other hand, from the descriptive results obtained for types of violence, it can be observed that the data on violence exercised and received by adolescents are low, and that the participants indicate more easily and frequently that they have been subjected to some type of violent behavior in contrast to having used it.

The results obtained for perceived violence show that the sample in general identifies behaviors as quite violent. However, there is a greater variation in the answers to this variable, since some higher standard deviations can be observed in all types of violence perceived.

With respect to the main research objective, in the analysis of the relation between Emotional Intelligence and violence exercised, Perceived and Received in dating relationships (see Table 2), only Violence Exercised and Perceived correlate with some of the dimensions of Emotional Intelligence. These relations are always in the same direction. On the one hand, all the dimensions of Violence Exercised relate inversely with the dimensions of Emotional Intelligence (the lower Emotional Intelligence is, the greater the Violence Exercised). On the other hand, all types of Violence Perceived correlate directly with Emotional intelligence (the greater Emotional Intelligence is, the greater the perception of Violence is).

The results for total violence show that violence exercised relates to the following dimensions: Interpersonal Intelligence, Adaptability, Stress Management, and total Emotional Intelligence. That is to say, the lower the score for these dimensions of Emotional Intelligence, the more violence is exercised. Perceived violence correlates with the following dimensions: Interpersonal Intelligence, Adaptability, and Total Emotional Intelligence. Therefore, the higher the score obtained for these variables, the greater the ability to identify violent behaviors is. More specifically and focusing on each type of violence, the results show the following:

The data indicate that there is a relation between Physical Violence exercised and skills in Stress Management and the Emotional Intelligence displayed by the subjects (the lower the ability to manage stress and the lower total emotional intelligence is, the greater the physical violence exercised. The data also show that there is a relation between Perceived Violence and *p* < 0.05 of Interpersonal Intelligence, Adaptability, and Total Emotional Intelligence (the higher the score in these variables, the greater the subjects’ ability to identify behaviors such as physical violence).

As with the previous variable, perceived Sexual Violence relates directly to the following variables: Interpersonal Intelligence, Adaptability, and total Emotional Intelligence. That is to say, the higher the score obtained in these variables, the greater the ability to identify behaviors of sexual violence. However, Sexual Violence exercised only relates to the dimensions of Interpersonal Intelligence and total Emotional Intelligence. Therefore, only these two dimensions seem to influence whether more or less sexual violence is exercised; so, the lower the score in these dimensions, the more sexual violence is exercised.

Unlike the other dimensions, Psychological Social Violence only relates directly to the dimensions of Emotional Intelligence when referring to perceived violence. This occurs specifically with Interpersonal Intelligence, Adaptability, and total Emotional Intelligence.

As with Physical Violence, Psychological Violence of Humiliation relates to the dimensions of total Emotional Intelligence and Stress Management. Therefore, the greater the score in these variables of Emotional Intelligence is, the lower the Psychological Violence of Humiliation is. On the other hand, perceived Psychological Violence of Humiliation presents a direct relation to the dimensions of Interpersonal Intelligence, Adaptability, and Total Emotional Intelligence.

Psychological Violence of Control exerted is the variable that relates most significantly to the different dimensions of Emotional Intelligence: Interpersonal Intelligence, Adaptability, Stress Management, and Emotional Intelligence. Thus, the lower the score in these dimensions, the more Psychological Violence of Control is used.

As with the other types of perceived violence, perceived Psychological Violence of Control relates significantly and directly to Interpersonal Intelligence, Adaptability, and total Emotional Intelligence. These three dimensions of Emotional Intelligence are the ones that are linked to a greater ability to identify the violent behaviors that occur in partner relationships.

After analyzing the relation between Emotional Intelligence and violence that adolescents exercise, receive, and perceive in dating relationships, several mean comparisons for independent samples were carried out using the Student’s T-test. The following results were obtained.

In the first specific objective, the comparison between boys and girls for their scores in the dimensions of Emotional Intelligence show significant differences in the ability to manage stress, which was higher in men than in women (see Table 3).

As shown in Table 4, with respect to the second and third specific research objectives, the comparison of the mean values obtained for the different types of violence exercised or received reflects a significant difference in Sexual Violence between men and women that have had at least one dating relationship. In this case, men obtained a higher score than women.

In the analysis of the fourth specific research objective (see Table 5), the only significant difference between boys and girls for Violence Perceived can be found in their scores for perception of sexual violence. The highest score was obtained by women, so the girls seem to be more easily able to identify behaviors of sexual violence as aggressive.

Finally, in Table 6, to analyze the fifth specific research objective, we carried out a mean comparison of the dimensions of violence perceived in those who have had a dating relationship and those who have not. Significant differences were found in all types of violence perceived, showing that those who have not had a dating relationship show a higher mean in all dimensions in contrast to those that have had a relationship. This indicates that those who have not had a dating relationship identify behaviors as more violent than those who have had a relationship, since they do not identify them as so violent.

## 4. Discussion

As indicated in previous studies, the results obtained confirm that violence in teenage dating relationships does occur, especially physical and psychological violence, which is occasionally exercised and received [7,9,11,12,13]. However, the data obtained show that adolescents have only slightly used or been subjected to the behaviors asked about in the questionnaire. As pointed out in different research papers [8,9,39], this data could indicate a certain permissiveness by adolescents towards the use of violence to solve conflicts, or that they are unable to acknowledge that certain actions between a couple are violent. For example, insults, blackmail, aggressions, or certain behaviors of vigilance and dominance etc.

A clear example of this can be observed in qualitative responses by both boys and girls to the evaluation instruments, where they indicate that behaviors such as biting, pushing, and looking at their partner’s Whatsapp messages are non-violent behaviors. They justify these behaviors by saying that they do it as a joke, or that looking at their partner’s things is normal because if their partner did not allow them to do it, it would mean they had something to hide [40,41,42].

The results obtained in violence perceived reinforce these observations since subjects are shown to generally perceive behaviors as only a little or quite violent. In fact, even when a comparison is made between the groups of subjects with or without a partner, those that have had a dating relationship are shown to perceive violent behaviors by partners as less aggressive than those who have not had a dating relationship. This could be due to the inexperience of adolescents, their lack of certain assertive skills related to communication and solving conflicts, low self-esteem, or being unaware of or having difficulty in detecting violent behavior.

It can be observed that adolescents within the group of people who have had a partner see themselves more as victims that receive violent behaviors than as aggressors who use violence against their partners [43]. As indicated in other studies [44,45], this could be due to the fact that because of their social desirability, adolescents do not want to manifest behaviors that would show that they use violence towards their partner or because they are unable to recognize that such behaviors are violent. For this reason, the Internet could be used in future research as a means of applying tests, since according to Sáenz [45], this method provides good results and reduces social desirability in subjects.

With respect to the differences between boys and girls, the results obtained indicate that girls have had a greater number of partner relationships than boys, and start having relationships at an earlier age than them. This can be explained by the fact that girls have an earlier maturation development than boys, since the onset of puberty in girls is between 9 and 11 years old, while for boys it is between 12 and 14 [46].

Differences between sexes in Emotional Intelligence cannot be affirmed conclusively, as found in other studies [47,48,49]. The only significant results are those obtained in the variable stress management, where the boys have obtained a higher score than the girls. This indicates that boys have more skills in stress management than girls.

For the type and frequency of violence adolescents use and receive, significant differences have only been found in the sexual violence exercised. In this case, the boys have obtained a higher score than girls, which is in line with other studies [21,22,26,27]. However, it is true that these scores are not very high and in the rest of violent behaviors, there are no significant differences between sexes. Therefore, it can affirmed that in general both girls and boys exercise and receive the same number of violent behaviors, although the frequency of these violent acts is not very high. This is also observed in other studies [20,28,43], where all adolescents, regardless of whether they are boys or girls, receive, exercise, and perceive the same amount of physical violence, psychological violence of control and humiliation, and sexual violence.

After observing all the results and according to other articles [20,28,43], the hypothesis established is therefore supported. That is to say, all adolescents regardless of whether they are girls or boys receive, exert, and perceive the same amount of physical violence, psychological violence of control or humiliation and sexual violence.

In the case of the relation between Emotional Intelligence and violence that adolescents exercise, receive and perceive in their dating relationships, it can be observed that there is a significant and inverse relationship with the violence that young people use. It also relates directly and significantly to the ability to perceive certain behaviors as violent. This shows that being the aggressor in adolescent dating relationships is related to obtaining lower scores in Emotional Intelligence, and that being able to identify violent behaviors as aggressive is related to higher scores in Emotional Intelligence.

These results are a clear example of how important it is to educate people in Emotional Intelligence from childhood within the family and school sphere. It is evident that Emotional Intelligence is related to these violent behaviors in dating relationships in young people and adolescents as well as to other violent behaviors like school bullying, criminal offences, anti-social behaviors, etc. [32]. Therefore, it is undoubtedly a predictor of violent behaviors and its development is essential to personal growth.

According to the results obtained in this study and on the basis of the above points and those presented in the introduction of this research paper, the hypotheses formulated can be accepted.

Emotional Intelligence will relate inversely and significantly to the violence that adolescents use and receive in their dating relationships.

Emotional Intelligence will relate inversely and significantly to the violence perceived by adolescents in their dating relationships.

The group of adolescents who have not had any dating relationships will obtain significantly higher scores in their perception of violent behaviors than those who have had/have a dating relationship.

With respect to the hypotheses that boys will show significantly higher scores in the use of physical and sexual violence compared to girls, the use of more sexual violence by boys can be confirmed. However, the assumption that they use more physical violence is not supported.

Likewise, the rest of the hypotheses formulated in this study are not confirmed since no significant differences are observed between the scores for men and women in Emotional Intelligence. Girls do not obtain higher scores than men in either the use of psychological violence or in victimization. In fact, the scores are similar for both sexes.

Finally, it is of interest to highlight some of the limitations in this study and in this way contribute to the improvement of future research in this field.

Firstly, as this is a transversal study carried out in an institute, it is not possible to establish conclusive data on violent behavior over time. This is due to the fact that the sample is not broad enough or representative of the population at an international, national, provincial, or local level. For this reason, it would be necessary to develop a study using longitudinal measures (over time) and including participants from different schools, areas, and countries.

Another aspect to be considered is that this research may contain a certain amount of bias. This could arise with respect to questions adolescents answer about whether certain behaviors are a little or very violent and whether they used or received them. In this sense, adolescents may feel that to a certain extent they are being judged and therefore answer with socially accepted responses, and consequently, the answers do not reflect the reality of the situations of dating relationships in adolescents. Thus, as mentioned above, it would be useful to provide a more individual way of completing the questionnaire, where the subjects do not feel they are being observed and judged by either the researchers or by their classmates. This could be done by using online questionnaires or by completing questionnaires individually rather than in a group.

In addition, throughout adolescence, different physical, cognitive, and social changes take place that can affect the use of these types of violent behaviors. Therefore, it would be of interest for future research to carry out a comparative analysis according to the age and school year of adolescents.

Finally, it should be mentioned that the magnitudes obtained for the relations between the different dimensions of Emotional Intelligence and behaviors of violence exerted, received, and perceived are not very high. Thus, it could be said that although it does seem to be a variable that has an important role in the development of adolescents and their violent and prosocial behaviors, it is not the only predictor of violent behaviors in adolescent dating relationships. This indicates that there is a need to continue research into discovering all those agents that represent risk or protective factors for adolescents to engage in such behaviors individually and when interacting with others.

## 5. Conclusions

It can be concluded from this study that Emotional Intelligence is an important factor in the violence that adolescents use, receive, or perceive. It is evident that high Emotional Intelligence leads to fewer violent behaviors being used in dating relationships and a greater ability to identify and perceive violent behaviors as aggressive. The most significant dimensions in these violent behaviors are: Interpersonal Emotional Intelligence, Adaptability, and Stress Management.

In addition, it can be observed that in this stage, there are no differences in Emotional Intelligence and violence that either men or women use, receive, and perceive. That is to say, both sexes have similar scores in the different dimensions and the total score for Emotional Intelligence, and they exercise, receive, and identify violent behaviors in adolescent dating relationships in a similar way.

In spite of its limitations, this study provides some interesting data about the importance of cultivating and educating both male and female adolescents in different skills, such as early prevention of dating violence. This could be done through primary and secondary prevention as a means of stopping them from becoming victims and aggressors in their relationships as adolescents, and consequently as adults. In general, tertiary prevention is carried out, but this is when people are already in the role of victim or aggressor in the relationship [7]. Moreover, this study reveals the important role that Emotional Intelligence has in these types of behaviors, and the need to provide education in this area in order to prevent these violent behaviors, and in this way develop personal growth which will lead to the improvement of partner relationships

This paper provides new data that contributes to the development of this specific line of research, which is violence in adolescent couples, and which, as indicated, has its own specific characteristics.

## Figures and Tables

**Table 1:** Descriptive analysis of Emotional Intelligence and types of violence.

	Mean	SD	*N*
Intrapersonal	13.64	3.554	254
Interpersonal	19.70	2.495	254
Adaptability	16.89	3.086	254
Stress management	16.29	3.373	254
Positive Impression	14.50	2.090	254
Total	81.03	8.062	254
Physical V. Received	0.50	0.716	172
Sexual V. Received	0.24	0.513	172
Social V. Received	0.23	0.447	172
V. of Humiliation Received	0.33	0.530	172
V. of Control Received	0.59	0.809	172
Physical V. Exercised	0.36	0.594	172
Sexual V. Exercised	0.06	0.200	172
Social V. Exercised	0.10	0.190	172
V. of Humiliation Exercised	0.15	0.233	172
V. of Control Exercised	0.37	0.584	172
Physical V. Perceived	2.52	0.978	254
Sexual V. Perceived	2.49	1.042	254
Social V. Perceived	2.68	1.048	254
V. of Humiliation Perceived	2.61	1.107	254
V. of Control Perceived	2.19	0.943	254
Total V. Received	0.38	0.473	172
Total V. Exercised	0.21	0.255	172
Total V. Perceived	2.49	0.924	254

**Table 2:** Relation between Violence and Emotional Intelligence.

			Intra-Personal	Inter-Personal	Adapt-Ability	Stress Manage-Ment	Positive Impression	Total EI
		Pearson Correlation	−0.53	−0.188 *	−0.178 *	−0.249 *	−0.192 *	−0.295 *
	Exercised	Sig.(bilateral)	0.493	0.013	0.019	0.001	0.012	0.000
Total		Pearson Correlation	0.007	0.198 *	0.205 *	0.085	0.038	0.188 *
	Perceived	Sig.(bilateral)	0.911	0.002	0.001	0.177	0.550	0.003
		Pearson Correlation	−0.061	−0.107	−0.126	−0.164 *	−0.106	−0.198 *
	Exercised	Sig.(bilateral)	0.427	0.161	0.099	0.031	0.168	0.009
Physical		Pearson Correlation	0.066	0.238 *	0.205 *	0.045	0.077	0.220 *
	Perceived	Sig.(bilateral)	0.292	0.000	0.001	0.476	0.222	0.000
		Pearson Correlation	0.73	−0.236 *	−0.136	−0.061	−0.150	−0.153 *
	Exercised	Sig.(bilateral)	0.341	0.002	0.074	0.426	0.050	0.000
Sexual		Pearson Correlation	−0.013	0.180 *	0.206 *	0.085	−0.008	0.162 *
	Perceived	Sig.(bilateral)	0.833	0.004	0.001	0.175	0.893	0.010
		Pearson Correlation	−0.044	0.198 *	0.183 *	0.079	0.015	0.149 *
Social	Perceived	Sig.(bilateral)	0.486	0.002	0.003	0.207	0.812	0.017
		Pearson Correlation	−0.069	−0.059	−0.003	−0.247 *	−0.187 *	−0.198 *
Humilia	Exercised	Sig.(bilateral)	0.372	0.444	0.968	0.001	0.001	0.009
tion		Pearson Correlation	−0.029	0.1620 *	0.202 *	0.068	0.071	0.162 *
	Perceived	Sig.(bilateral)	0.646	0.010	0.001	0.280	0.260	0.010
		Pearson Correlation	−0.059	−0.164 *	−0.180 *	−0.216 *	−0.147	−0.265 *
	Exercised	Sig.(bilateral)	0.442	0.032	0.018	0.004	0.054	0.000
Control		Pearson Correlation	0.064	0.128 *	0.124 *	0.102	0.018	0.163 *
	Perceived	Sig.(bilateral)	0.308	0.041	0.048	0.105	0.777	0.009

* Note: This coefficient is significant (*p* < 0.05).

**Table 3:** Differences between men and women in Emotional Intelligence.

Emotional	Sex	Mean (DT)	t-Student		Size
Intelligence			t	*p*	Effect (r)
Intrapersonal	Man	13.74 (3.532)			
			0.454	0.651	0.07
	Woman	13.53 (3.563)			
Interpersonal	Man	19.39 (2.580)			
			−1.675	0.095	0.11
	Woman	19.93 (2.434)			
Adaptability	Man	16.78 (3.234)			
			−0.314	0.754	0.02
	Woman	16.90 (3.003)			
Stress management	Man	17.49 (2.769)			
			5.105	0.000 *	0.31
	Woman	15.46 (3.475)			
Positive Impression	Man	14.64 (2.025)			
			0.682	0.496	0.04
	Woman	14.45 (2.116)			
Total Intelligence	Man	82.03 (8.142)			
			1.693	0.092	0.11
	Woman	80.28 (8.013)			

* Note: This coefficient is significant (*p* < 0.05).

**Table 4:** Differences in Violence Exercised and Received according to sex.

Type of Violence	Sex	Mean (SD)	t−Student		Effect
			t	*p*	Size (r)
Physical V. Exercised	Man	0.25 (0.411)			
			−1.826	0.070	0.14
	Woman	0.41 (0.680)			
Sexual V. Exercised	Man	0.11 (0.290)			
			2.080	0.041 *	0.024
	Woman	0.03 (0.104)			
Social V. Exercised	Man	0.09 (0.185)			
			−0.346	0.729	0.03
	Woman	0.10 (0.194)			
V. of Humiliation	Man	0.10 (0.200)			
Exercised			−1.959	0.052	0.16
	Woman	0.17 (0.250)			
V. of Control	Man	0.32 (0.418)			
Exercised			−0.780	0.436	0.06
	Woman	0.39 (0.676)			
Physical V. Received	Man	0.60 (0.802)			
			1.748	0.082	0.14
	Woman	0.41 (0.595)			
Sexual V. Received	Man	0.28 (0.595)			
			0.848	0.398	0.07
	Woman	0.21 (0.466)			
Social V. Received	Man	0.27 (0.512)			
			0.814	0.417	0.06
	Woman	0.21 (0.414)			
V. of Humiliation	Man	0.42 (0.500)			
Received			1.731	0.716	0.013
	Woman	0.27 (0.553)			
V. of Control	Man	0.66 (0.845)			
Received			0.973	0.332	0.08
	Woman	0.54 (5.786)			
Total V. Exercised	Man	0.18 (0.289)			
			−1.157	0.249	0.09
	Woman	0.22 (6.162)			
Total V. Received	Man	0.45 (0.513)			
			1.569	0.119	0.12
	Woman	0.33 (0.443)			

* Note: This coefficient is significant (*p* < 0.05).

**Table 5:** Differences in Violence Perceived according to sex.

Type of Violence	Sex	Mean (SD)	t-Student		Effect
			t	*p*	Size (r)
Physical V. Perceived	Man	2.46 (0.902)			
			−0.840	0.402	0.05
	Woman	2.57 (1.044)			
Sexual V. Perceived	Man	2.33 (1.006)			
			−2.059	0.041 *	0.13
	Woman	2.61 (1.055)			
Social V. Perceived	Man	2.61 (0.971)			
			−0.766	0.444	0.05
	Woman	2.71 (1.114)			
V. of Humiliation	Man	2.48 (0.936)			
Perceived			−1.652	0.100	0.10
	Woman	2.71 (1.222)			
V. of Control	Man	2.07 (0.911)			
Perceived			−1.602	0.110	0.10
	Woman	2.26 (0.961)			
Total V. Perceived	Man	2.38 (0.845)			
			−1.582	0.115	0.10
	Woman	2.57 (0.981)			

* Note: This coefficient is significant (*p* < 0.05).

**Table 6:** Differences in Violence Perceived between people who have had a dating relationship and those who have not.

Type of Violence	Yes/No	Mean (SD)	t-Student		Effect
	Partner		t	*p*	Size (r)
Physical V. Perceived	Has not had	2.96 (0.678)			
			6.044	0.000 *	0.37
	Has had	2.31 (1.030)			
Sexual V. Perceived	Has not had	2.90 (0.754)			
			5.151	0.000 *	0.33
	Has had	2.29 (1.105)			
Social V Perceived.	Has not had	3.23 (0.721)			
			7.168	0.000 *	0.43
	Has had	2.41 (1.079)			
V. of Humiliation	Has not had	3.09 (0.646)			
Perceived			6.128	0.000 *	0.36
	Has had	2.38 (1.207)			
V. of Control	Has not had	2.66 (.661)			
Perceived			6.787	0.000 *	0.41
	Has had	1.96 (0.974)			
Total V. Perceived	Has not had	2.96 (0.845)			
			7.027	0.000 *	0.42
	Has had	2.526 (0.981)			

* Note: This coefficient is significant (*p* < 0.05).

## Data Availability

The data presented in this study are available from the corresponding author on reasonable request.
